# Editorial: Rhizosphere microbiome engineering for crop cultivation

**DOI:** 10.3389/fbioe.2023.1267442

**Published:** 2023-08-29

**Authors:** Xu Cheng, Mengcen Wang, Mengting Maggie Yuan, Jiangang Li, Wu Xiong

**Affiliations:** ^1^ Shenzhen Branch, Guangdong Laboratory of Lingnan Modern Agriculture, Key Laboratory of Synthetic Biology, Ministry of Agriculture and Rural Affairs, Agricultural Genomics Institute at Shenzhen, Chinese Academy of Agricultural Sciences, Shenzhen, China; ^2^ State Key Laboratory of Rice Biology, Ministry of Agricultural and Rural Affairs Laboratory of Molecular Biology of Crop Pathogens and Insects, Zhejiang University, Hangzhou, China; ^3^ Department of Environmental Science, Policy and Management, University of California, Berkeley, Berkeley, CA, United States; ^4^ Institute of Soil Science, Chinese Academy of Sciences, Nanjing, China; ^5^ Jiangsu Provincial Key Lab for Organic Solid Waste Utilization, National Engineering Research Center for Organic-based Fertilizers, Jiangsu Collaborative Innovation Center for Solid Organic Waste Resource Utilization, Nanjing Agricultural University, Nanjing, China

**Keywords:** rhizosphere, microbiome, crop, disease suppression, growth and resilience

## Introduction

Crops in natural environments encounter a wide range of abiotic and biotic stresses, which consequently limit their growth and yield, posing a significant threat to global food security. The myriad of rhizosphere microorganisms inhabiting the interface between plant roots and soil can serve to improve plant health by contributing to enhanced immunity, stress resilience and nutrient acquisition. Rhizosphere microbiome engineering allows for the rational utilization of the potential functions within microbial communities, enabling stable and high-yield crop production. More than ever, it has been regarded as an effective solution to address the global challenges posed by exponential population growth with limited food production.

## Rhizosphere microbiome

The concept of “rhizosphere” was first proposed by Lorenz Hiltner in 1904. It was defined as a highly dynamic and complex soil microenvironment directly influenced by plant roots, with unique biological and soil physicochemical characteristics. The rhizosphere is a critical niche for the interaction among plants, soil, and microorganisms. Plants coevolved with microbes and rely on them for nutrient acquisition and protection against various abiotic and biotic stresses. Environmental constraints such as abiotic stress from drought, soil salinity and sodicity, as well as biotic stress from pests and pathogens, pose significant challenges to agricultural production, resulting in crop yield reduction and quality losses. Designing and maintaining a well-balanced rhizosphere microbiome using appropriate tools has emerged as a promising strategy to enhance nutrient uptake efficiency and improve resistance against pests and pathogens, which holds crucial significance for the modern sustainable agriculture.

## Rhizosphere microbiome engineering

With the continuous innovation of tools and methods, the manipulation and management of the rhizosphere microbiome has gradually become proficient and sophisticated. Traditional pathways for the rhizosphere microbiome engineering include direct and indirect approaches. Direct rhizosphere microbiome engineering refers to the application of beneficial microbial inoculants. Beneficial microorganisms, such as plant growth-promoting rhizobacteria (PGPR) and arbuscular mycorrhizal fungi (AM), have been widely employed as bioinoculants in agricultural production. However, most beneficial strains were isolated in laboratory. They demonstrated growth promoting or disease suppressive effects under sterile conditions or in pot experiments but fail to replicate their performance when applied in field conditions due to the failure to compete with the local microbiome.

Indirect rhizosphere microbiome engineering mainly focuses on using agricultural practice (changing cultivation pattern: such as crop rotation or intercropping) and addition of soil amendments to alter rhizosphere environment for the manipulation of the rhizosphere microbiome. These methods can improve soil physicochemical properties, indirectly enhance soil activity, and induce the formation of suppressive soils. Nevertheless, the mechanisms that drive these functional microbial community assembly cannot be clearly explained. Soil amendment application may also cause accumulation of toxic substances that may harm the soil microbiota.

With the ongoing development of tools and technologies, a large number of novel approaches for manipulating rhizosphere microbial communities have emerged. These novel approaches include customized functional microbial consortia (SynCom: synthetic microbial community), shaping the rhizosphere microbiome by crop-breeding or genetic modification, and improving soil environment by using root exudates to recruit beneficial bacteria. These novel engineering optimize the interaction between beneficial microbes and crops, enhancing the effectiveness of microbial promotion for stable and high-yield crop production.

## Current achievements

Over the past 2 years, a large number of research articles related to rhizosphere microbiome engineering have been published, reflecting the key technologies and latest achievements in manipulating the rhizosphere microbiome to promote stable and high-yield crop production. Zhou et al. (2022) successfully enhanced the suppressive ability of tomatoes against Fusarium wilt disease by constructing a cross-kingdom synthetic microbiota. Hong et al. (2023) demonstrated that crop rotation can assemble distinct core microbiota as functionally specific barriers against the invasion of pathogens. Their study provided theoretical support for identifying rotation crop-unique antagonistic taxa, and emphasized the importance of targeted cultivation of beneficial microorganisms in support of agriculture sustainability. Zhou et al. (2023) revealed a novel mechanism in which intercropped plants regulate inter-specific interactions through specific root exudates to establish disease-suppressive rhizosphere microbial communities.

Some key results were published in this Research Topic, including five original research articles, and a review. Park et al. addressed the intricate interplay among factors driving root microbiome assembly, and how it facilitates the recruitment of specific microbes by the host plant to support plant growth and resilience under stress. This review also highlighted that the rational manipulation of rhizosphere microbiome is a promising avenue to improve the quality and productivity of crops. Chen et al. reported how the maize-peanut rotation enriched certain PGPR such as *Bacillus*, *Streptomyces*, *Rhizobium*, and *Pseudomonas*, which protected root development and subsequently increased maize yield. Li et al. confirmed the correlation between sugarcane root rot severity and rhizosphere soil microbiome composition and function. They also isolated several biocontrol strains with disease suppressive effect and growth promoting characteristics. Zhao et al. demonstrated that the application of broccoli residues promoted the recruitment of beneficial bacteria in the cotton rhizosphere, which increased bacterial diversity, and significantly reduced the relative abundance of *V. dahlia*. Wei et al. investigated the response of soil microbial communities to drought stress under four different crop rotation systems in the western foot of the Greater Khingan range. Their study evidenced that soil organic matter was the main factor influencing changes in microbial community structure. Lu et al. illustrated that the occurrence of wheat yellow mosaic virus diseases and the different niches both altered fungal community diversity and composition. Together, all of these articles emphasized the key role of rhizosphere microbiome engineering in improving crop yield and quality.

## Outlook

In the coming decades, we will systematically and purposefully modify the root zone to fulfill the needs of sustainable agriculture. By enhancing the interaction and cooperation between beneficial microbes and indigenous microorganisms, we hope to design and construct the powerful, stable and functionally diverse rhizosphere microbiome. We look forward to further research that integrates emerging technologies such as synthetic biology, CRISPR, and multi-omics analysis, etc. These will help us deeply understand and fully utilize the positive plant-microbe interactions and develop the so-called “microbiome-driven cropping systems” in a near future.
